# A novel variant of *IHH* in a Chinese family with brachydactyly type 1

**DOI:** 10.1186/s12881-020-01000-6

**Published:** 2020-03-24

**Authors:** Qi Yang, Jin Wang, Xiaoxian Tian, Fei Shen, Jing Lan, Qiang Zhang, Xin Fan, Shang Yi, Mengting Li, Yiping Shen

**Affiliations:** 1 Genetic and Metabolic Central Laboratory, Birth Defect Prevention Research Institute, Maternal and Child Health Hospital, Children’s Hospital of Guangxi Zhuang Autonomous Region, Nanning, 530002 China; 2 Department of Ultrasonography, Maternal and Child Health Hospital, Children’s Hospital of Guangxi Zhuang Autonomous Region, Nanning, 530002 China; 3 Department of Gynaecology, Maternal and Child Health Hospital, Children’s Hospital of Guangxi Zhuang Autonomous Region, Nanning, 530002 China; 4 Department of Medical Genetics and Molecular Diagnostic Laboratory, Shanghai Children’s Medical Center, Shanghai Jiao Tong University School of Medicine, Shanghai, 200127 China; 5 Division of Genetics and Genomics, Boston Children’s Hospital; Department of Neurology, Harvard Medical School, Boston, MA 02115 USA

**Keywords:** Brachydactyly type A1, *IHH* gene, Variant

## Abstract

**Background:**

Brachydactyly type A1(BDA-1) is an autosomal dominant disorder which is caused by heterozygous pathogenic variants in a specific region of the N-terminal active fragment of Indian Hedgehog (*IHH*). The disorder is mainly characterized by shortening or missing of the middle phalanges. In this study, Our purpose is to identify the pathogenic variations associated with BDA-1 involved in a five-generation Chinese family.

**Methods:**

A BDA-1 family with 8 affected and 14 unaffected family members was recruited. Whole exome sequencing (WES) was performed to identify the pathogenic variant in the proband, and which was later confirmed and segregated by Sanger sequencing. The significance of variants were assessed using several molecular and bioinformatics analysis methods.

**Results:**

We uncovered a novel heterozygous missense variant c.299A > G (p.D100G) at the mutational hotspot of *IHH* gene following whole-exome sequencing of a Chinese family with BDA-1. The variant co-segregated with BDA-1 in the pedigree, showed 100% penetrance for phalange phenotype with variable expressivity.

**Conclusions:**

In conclusion, this study reports a five-generation Chinese family with BDA-1 due to a novel pathogenic variant (c.299A > G (p.D100G)) of IHH and expands the clinical and genetic spectrum of BDA-1.

## Background

Brachydactyly (BD) is generally characterized by shortened and often malformed digits of the hands [1]. Heritable BDs have been classified into seven types, i.e.: A1, A2, A3, B, C, D, and E on the basis of their patterns of skeletal involvement [2]. Brachydactyly A-1 (BDA1; MIM 112500) is inherited as an autosomal dominant disorder and is characterized by short stature and shortening of middle phalanges of all the digits. The middle phalanges are either rudimentary or fused with the terminal phalanges. About half of the BDA1 families are due to mutations in the *IHH* (Indian Hedgehog) gene [3]. To date, about 14 different *IHH* pathogenic variants had been identified in individuals with BDA1 (HGMD and ClinVar), and the pathogenic variants cluster in the central region of the N-terminal signaling fragment [4]. As a central signaling molecule in mediating skeletal development, IHH plays an important role in mediating skeletal condensation, growth and differentiation of chondrocyte, joint development and bone formation [5]. Here, we studied a five-generation Chinese family associated with a variation of BDA1 and identified a novel *IHH* pathogenic variant by whole-exome sequencing.

## Materials and method

### Subjects

**Fig. 1:**
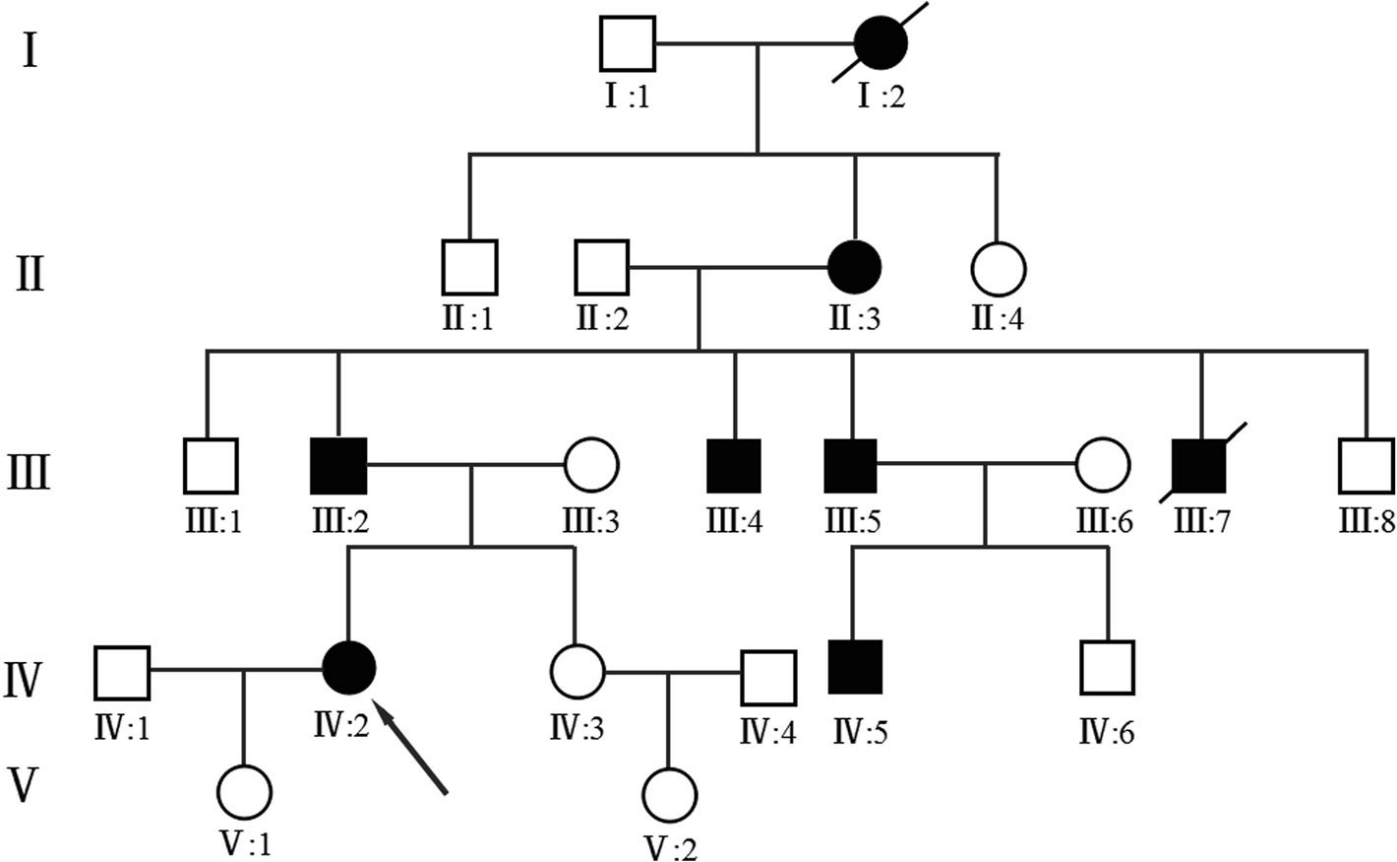
Pedigree of a five generation family with brachydactyly type A1 (BDA1). Filled symbols represent affected individuals; open symbols unaffected individuals; squares depict males and circles females. Diagonal lines indicate deceased individuals. The proband is indicated by an arrow

The BDA-1 affected family was referred to Guangxi Maternal and Child Health Hospital for shortened and malformed digits and requested genetic testing for all 22 family members. Diagnosis was based on physical examination, radiographic findings and family history. There were 20 individuals tested in the BDA1-affected family including 6 individuals who were diagnosed with BDA1. In order to rule out the possibility that the variant is unique to the region, we recruited 200 local residents for alternative allele frequency testing in 2018. The control group consisted of 100 females and 100 males aged between 20 to 40. The height of each individual in the control group was taller than 160 cm. The fingers and toes of the control group appears normal. All participants recruited in this study provided informed consent for the study approved by the ethics committee of the Maternal and Child Health Hospital of Guangxi Zhuang Autonomous Region. The pedigree was shown in Fig. 1.

### Genetic analysis

#### Whole-exome sequencing and sanger sequencing

Genomic DNA was extracted from peripheral blood using standard protocols of Lab-Aid DNA kit (Zeesan Biotech Co., Ltd., Xiamen, China). Agilent SureSelect Human All Exon V5 Kit (Agilent Technologies, Santa Clara, CA) was used for target capture. The library was sequenced on the Hiseq 2500 platform (Illumina, San Diego, CA, USA) according to the manufacturer’s instructions. A custom pipeline mainly built on the Genome Analysis Toolkit (GATK) was used for sequence data analysis and annotation. Identification of causal variant was aided by the TGex software (LifeMap Sciences,USA). We performed genotyping on 200 subjects by Sanger sequencing.

CADD (https://cadd.gs.washington.edu/snv; > 20 Damaging, ≤2 Tolerable 0)), SIFT (http://sift.jcvi.org/; < 0.05 deleterious, ≥0.05 tolerated), PolyPhen2 (http://genetics.bwh.harvard.edu/pph2/; 0–0.15 Benign, 0.15–0.85 Possibly damaging, 0.85–1 Probably damaging) and MutationTaster (http://www.mutationtaster.org/; disease-causing, polymorphism) were used to analyze the functional effects of novel variants with their respective cut-offs. Three-dimensional structures of wild type (WT) and mutant (MUT) IHH were predicted using SWISS-MODEL (https://swissmodel.expasy.org/interactive) by importing WT and MUT-IHH amino acid sequences. The candidate IHH variant was validated by Sanger sequencing and its pathogenicity classified following ACMG/AMP Guidelines [6].

## Results

### Clinical phenotype

**Fig. 2:**
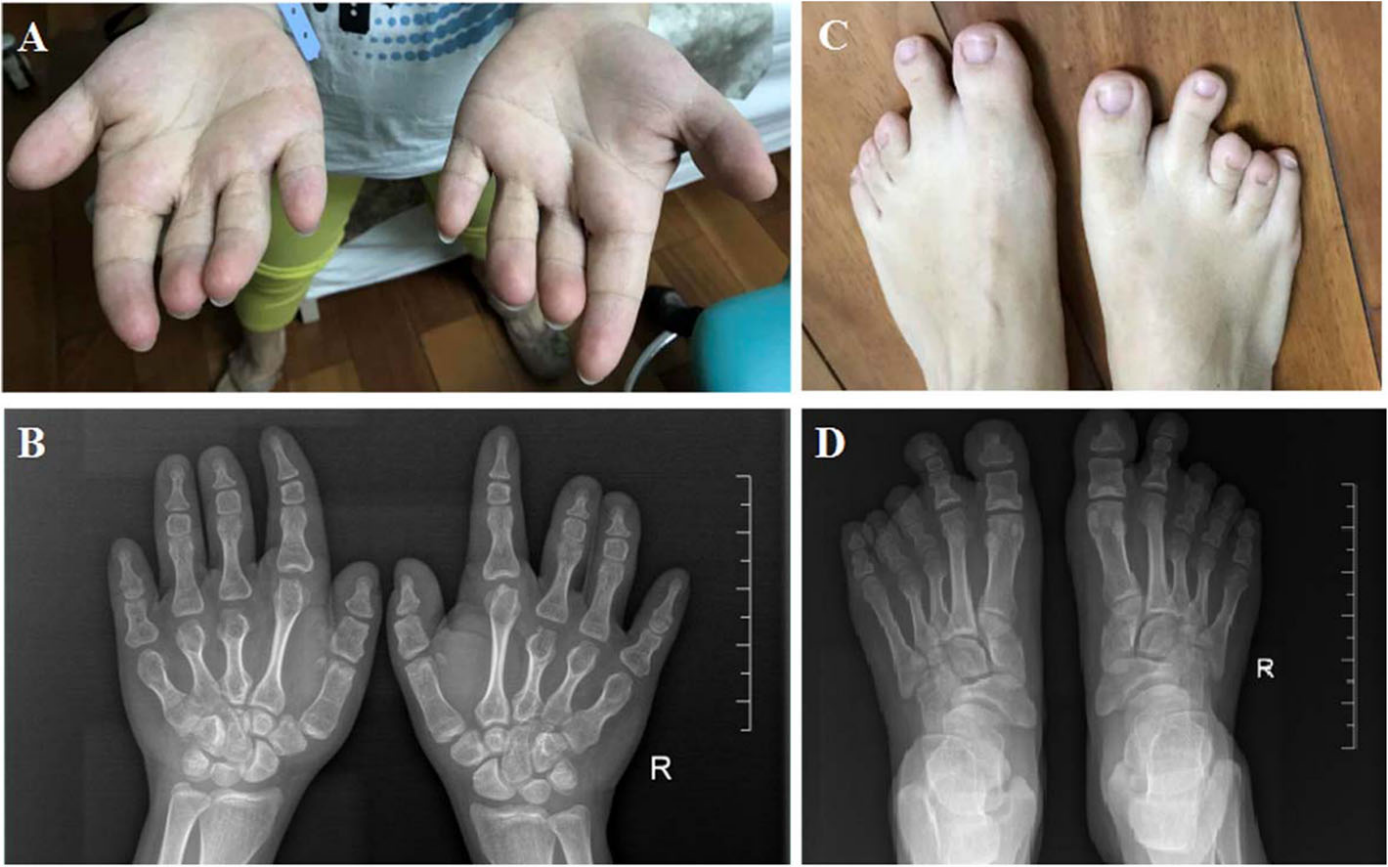
**a–d** The appearance and radiological findings of the proband with brachydactyly type A1(BDA1). **a** showing shortened fingers and absence of middle phalanges of the fifth finger and radial deviation of the second finger. **b** Radiographic images of proband’s hand: shortening of the middle phalanges of digits II–V, fusion of middle and terminal phalanges of 5th finger, bilateral shortening of metacarpals bone 3–5. **c** showing short toes and absence of middle phalanges of the third to fifth toes. **d** Radiographic images of proband’s foot: fusion of middle and terminal phalanges of third to fifth toe, bilateral shortening of metatarsals bone 3–4

**Fig. 3:**
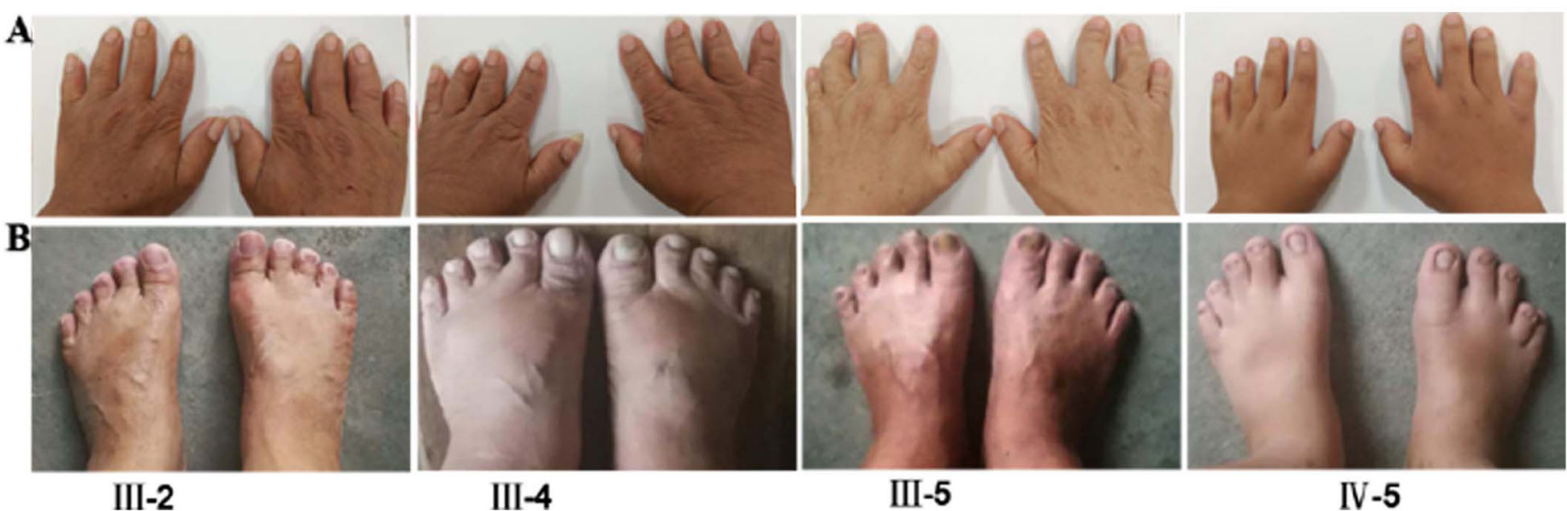
Features of other affected family members with brachydactyly type A1 (BDA1). **a** showing shortened fingers and radial deviation of the second or/and third finger (III-2, III-5, IV-5) and flexion contracture of the 4nd finger (III-5). **b** showing abnormally shortened toes

We constructed a pedigree of the five-generation family that participated in this study, which includes 6 family members affected by BDA-1, 2 affected members have been deceased, and 14 family members who are unaffected (Fig. 1,Table 1). We identified a *IHH* variant c.299A > G / p.Asp100Gly which co-segregate with BDA-1 phenotype in this family. Based on the number of affected individuals in this family, the LOD score was 1.5 which provided the supportive evidence for the pathogenicity (PP1_Strong) according to the ACMG / AMP guidelines. Statistical test for the mean digit length cannot be performed because of the lack of standard reference for fingers and toes of Chinese population. Finger 2 and 3 revealed that the affected individuals had relatively shortened fingers and toes to varied degree. We compared the height of each family members to the height growth chart for Chinese population. Non-affected individuals had normal stature. Some of the affected individuals had normal stature and some have short stature. The proband (IV-2) was a 30-year-old female, who presented with mild disproportionate short stature with a Height Standard Deviation Score (HSDS) of − 2.4SD. The radiograph of her hands showed varying degrees of shortening of the middle phalanx of the second to fifth fingers, and the middle phalanges in digit five was fused to the terminal phalanx as only one interdigital joint was visible. She also showed bilateral shortening of metacarpals bone 3–5. The radiograph of her feet showed shortening of all digits, the middle phalanges of third to fifth toe were fused to the terminal phalange (Fig. 2). She also had bilateral shortening of metatarsals bone 3–4. All of the other affected family members exhibited features consistent with BDA1. The toes of other affected individuals in the family were severely shortened and so were fingers (Fig. 3). Interestingly, short stature was not consistently presented among the affected individuals. The proband’s uncle (III-4) and cousin (IV-5) presented with mild disproportionate short stature with a HSDS of − 2.4SD ± 0.3, but her father’s (III-2) and uncle’s (III-5) heights were within normal range. In addition, the proband (IV-5), her father (III-2) and cousin (III-5) showed radial deviation of the second finger (Fig. 3a: III-2 and III-5). The uncle (III-5) showed radial deviation the 2nd and 3nd finger and flexion contracture of the 4nd finger (Fig. 3a). No other abnormalities were observed in the affected family members.

**Table 1:** Clinical features of the patients with de novo IHH mutations

Patient	Gender	Age at last Examination (years)	Height	Common features	Additional features
III-2	Male	50y	168 cm (normal)	Shortened fingers and toes	–
III-4	Male	49y	157 cm(<−2.4SD)	Shortened fingers and toes	Radial deviation of the third finger,radial deviation of the second and third finger
III-5	Male	47y	169 cm (normal)	Shortened fingers and toes	Flexion contracture of the 4nd finger
IV-2	Female	30y	154 cm(<−2.4SD)	Shortened fingers and toes, absence of middle phalanges of the fifth finger, absence of middle phalanges of the third to fifth toes, fusion of middle and terminal phalanges of third to fifth toe,	Radial deviation of the second fingerbilateral shortening of metacarpals bone 3–5, bilateral shortening of metatarsals bone 3–4
IV-5	Male	19y	155 cm(<−2.5SD)	Shortened fingers and toes,absence of middle phalanges of the fourth toes.	Radial deviation of the second finger,

### Genetic analysis of whole exome sequencing

**Fig. 4:**
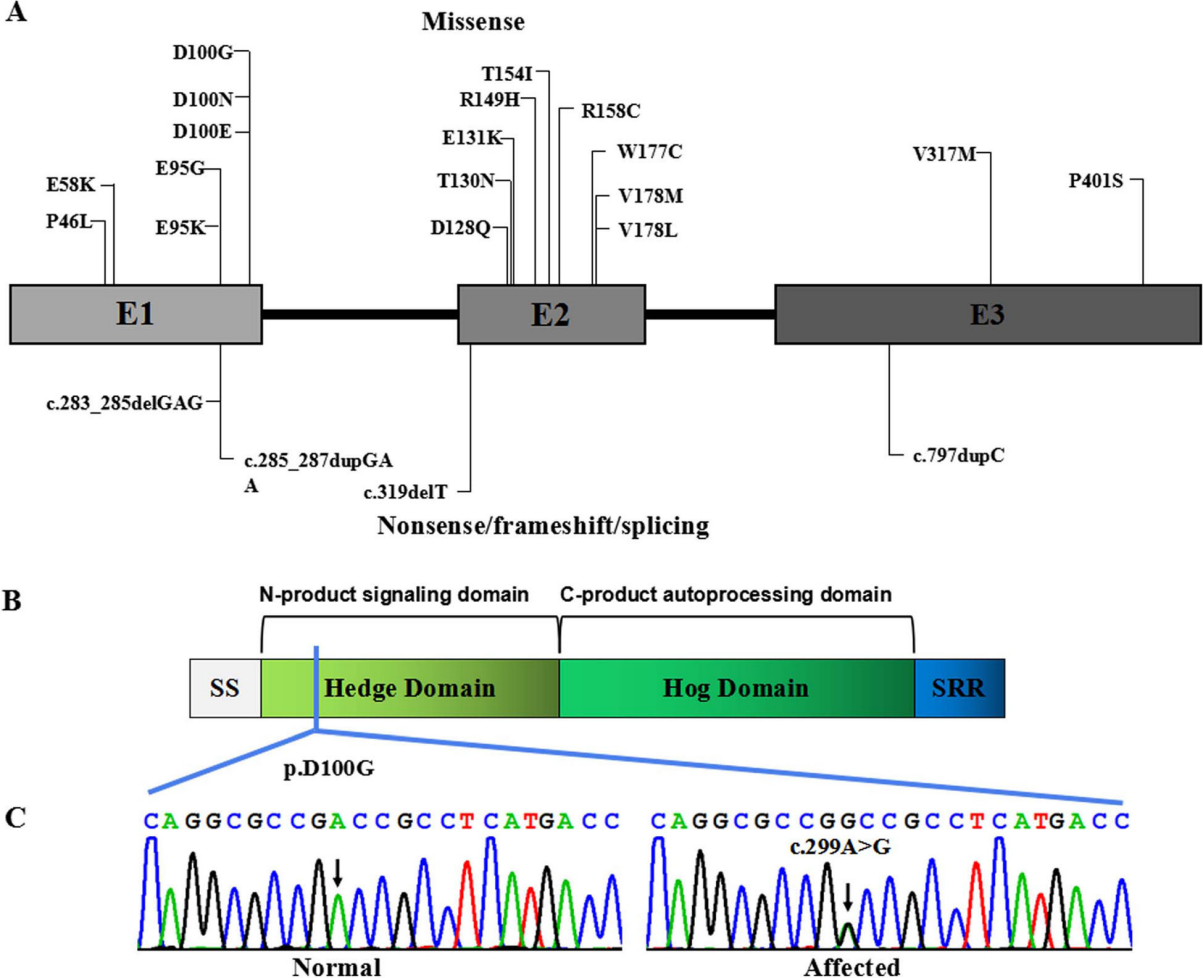
*IHH* pathogenic variants. **a** Boxes represent three different exons as indicated, and solid lines connecting these boxes represent the introns of *IHH* gene. The numbers above the boxes indicate the positions of the IHH complementary DNA at the start–stop sites and exon–intron boundaries. Vertical lines represent the locations of missense (above the boxes) or nonsense/frameshift/splicing (below the boxes) variants. **b** IHH protein structure with key domains, regions, and the mutation indicated. **c** Sanger sequencing chromatograms showing a missense variant c.299A > G(p.Asp100Gly) in the affected individuals in comparison to those of unaffected individuals

Whole exome sequencing using the genomic DNA of proband IV-1(Fig. 1) was performed. In total, 850 million uniquely mapped reads with MAPQ ≥30 were generated which covering 96% of exome target regions at least 20x. Exome sequencing called a total of 120,764 variants which excluded variants in non-functional variants such as intronic changes. A total of 16,040 variants were found in exonic and splice site regions. Among these variants, 1009 variants had the MAF less than 0.01 then neutral and benign variants were also omitted according to ClinVar. Based on the TGex software (LifeMap Sciences, USA), we found 17 variants existed in genes whose functions matched with known phenotypes. Variants from 6 genes associated with *IHH*, *NOTCH1*, *HMN*, *ATRX*, *BBS1* and *L1CAM* were extracted, leading to the identification of a novel variant in *IHH* c.299A > G / p.Asp100Gly that co-segregated with the disease phenotype in the examined family (Fig. 4c).

**Fig. 5:**
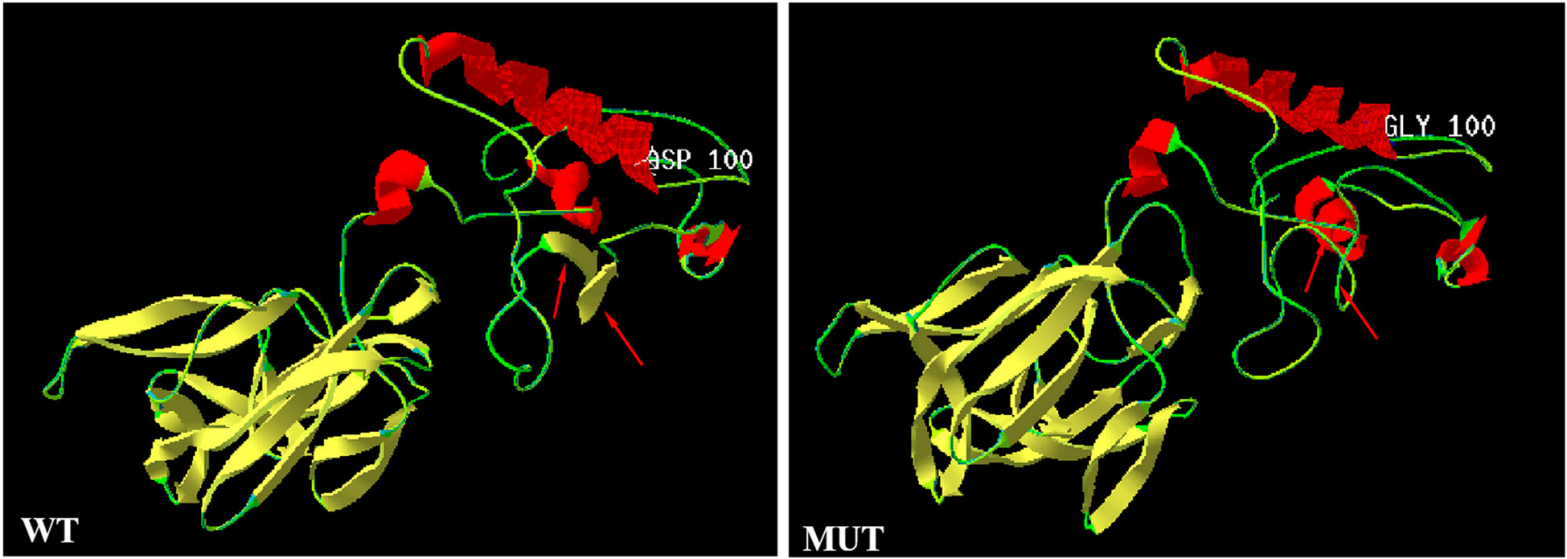
Three-dimensional structure modeling predicted a decrease in the proportion of β-sheet regions, and an increase of random coil regions in mutant protein. The dimer alterations are indicated by an arrow. WT, wild-type; MUT, mutant-type

The variant is not present in the Human Gene Mutation Database (http://www.hgmd.cf.ac.uk/ac/), HPSD (http://liweilab.genetics.ac.cn/HPSD/) and the dbSNP (http://www.ncbi.nlm.nih.gov/SNP/), nor it is present in the DNA samples from 200 normal or in the control databases (e.g. ExAC and gnomAD). The variant is located in N-terminal signaling domain as the DD-peptidase zinc-binding domain of IHH protein. The functional predictions for c.299A > G / p.Asp100Gly were as following: CADD score = 31 (Damaging), SIFT score = − 5.58 (Deleterious), PolyPhen2.0 score = 1 (Probably damaging) and Mutation Tasters (disease-causing). Further 3D structures of the wild type (WT) and mutant IHH were predicted using SWISS-MODEL (https://swissmodel.expasy.org/interactive). The result showed that the proportion of β-sheet would decrease and that of random coil increase in mutant protein compared to wild protein. In particular, domains 144–146 and 149–151 were predicted to be altered from β-sheets into random coils. The overall shape of IHH protein would be altered as shown in Fig. 5 which would consequently lead to reduced PTC1 binding and downstream signalling. It is revealed that the novel missense variant had deleterious effects. According to the AMP/ACMG guidelines for the interpretation of sequence variants [6], the novel variant is pathogenic (PM1, PM2, PM5, PP1_Strong, PP3, PP4).

## Discussion

Brachydactyly type A1 is characterized by hypoplasia/aplasia of the middle phalanges of digits 2–5. *IHH* was the first identified gene to be associated with BDA1 [7]. The *IHH* gene, which encodes a signaling protein of the Hedgehog family, is known for its role in endochondral ossification: regulate the balance between growth and ossification of developing bones [5]. The IHH protein operates through a feedback control mechanism where IHH binds to the patched (PTC) receptor which functions in association with smoothened (SMO), in order to activate the GLI complex of transcription factors [8–10]. From there, these transcription factors continue to signal and regulate down-stream genes affecting patterning.

*IHH* variants affect Hh signaling at multiple levels. It impairs chondrocyte maturation and proliferation, resulting in failure of osteoblast development in endochondral bones [7]. So far, about 14 *IHH* pathogenic variants have been reported to be associated with BD [11–14] (Fig. 4). All of the pathogenic variants are restricted to the N-terminal active fragment yet exhibit a variable outcome [4]. Variants associated with brachydactyly type A1 are known to affect codons 95, 100, 131, and 154 predominantly [3, 15, 16]. Based on the X-ray crystal structure of IHH, McLellan, et al. showed that these residues are located within a calcium binding site, an important domain for mediating interactions with PTCH1, HIP1, CDO and GAS1 [17]. Previous studies showed that p.D100E affected the IHH interactions with PTCH1 and HIP1 which resulted in reduced capacity to induce cellular differentiation [16], and p.D100N changed the Hh local tertiary structure and intracellular fate [5], causing abnormal bone development and abnormal digit formation. The protein 3D modelling prediction of the new mutant (p.D100G) also suggested an overall structural alteration, thus we suggest that the new mutant acted similarly to the p.D100E and p.D100N mutants which would affect Hh signaling at multiple levels, causing abnormal bone development and abnormal digit formation.

To date, six other BDA1-affected families of Italian, American, India, British and Chinese descent have been found to affect the same *IHH* codon at the position 100, demonstrating a mutational hot spot of *IHH*. The novel variant at the nucleotide position c.299 A > G of the *IHH* gene results in a novel amino acid substitution (p.D100G) at the hotspot. This novel variant co-segregated with the BDA1 phenotype in this Chinese family and demonstrated high penetrance of this pathogenic variant in causing dactyl phenotypes. In addition, phenotypic variations were observed among affected family members in terms of the severity of affected phalanges and metacarpal/metatarsal bones, demonstrating considerable intra-familial phenotypic variability.

IHH is expressed in the prehypertrophic chondrocytes of cartilage, and regulates growth of bones by coordinating chondrocyte proliferation and differentiation [18]. Dysregulation of IHH could affect the growth of phalangeal bones, as well as the long bones which could subsequently affect body height.

Although short stature is often a component of brachydactyly, it has been reported infrequently in type A1. It was only presented in brachydactyly type A1 patients with the variants at *IHH* Asp100 [3, 19, 20]. Interestingly, Gao et al. reported that all affected subjects were shorter than unaffected subjects in the same family whereas short stature was only presented for some family members reported by Giordano et al. and McCreadyet et al. [3, 19, 20], suggesting an incomplete penetrance of short stature associated with IHH variants. Consistently, in this Chinese family with a novel variant at residue 100, short stature was not 100% penetrant: the proband (IV-2), proband’s uncle (III-4), and cousin (IV-5) had short stature, whereas her father (III-2) and uncle (III-5) had normal stature. Pedigree studies provided the opportunity to identify co-determinants for human height. Gabriela et al. observed that heterozygous deleterious *IHH* variants are more frequent in short stature cohort (1.6%) in Brazilian and Spanish populations compared to the general population (0.017% in gnomAD; *P* < 0.001) [20], supporting the notion that reduced IHH signaling may be responsible for a reduced growth of the long bones and short stature [3, 7]. We suggest that the variant on Asp100 would reduce IHH signaling capacity which consequently reduced the growth of the long bones, and resulting in short stature. Certainly, there are other factors involved in affecting the final stature of individuals with *IHH* pathogenic variants. The role of *IHH* variants in non-syndromic short stature also needs further study.

## Conclusions

In conclusion, a novel missense variant (c.299 A > G) affecting a mutational hotspot (residue 100) of *IHH* was identified in a Chinese family affected by BDA-1. Sufficient evidence supported the pathogenicity of this novel variant. High penetrance for the phalange phenotype and variable expressivity were observed in this family. Short stature was only observed in a subset of affected family members. The findings of this report will further help our understanding the phenotype-genotype correlations of *IHH* pathogenic variants and related disorders including brachydactyly type A1.

## Data Availability

The data used and/or analyzed in the present report were deposited in the Sequence Read Archive (SRA) database. The data are accessible via the SRA accession: PRJNA612182; or via the links: https://www.ncbi.nlm.nih.gov/sra/PRJNA612182; https://trace.ncbi.nlm.nih.gov/Traces/sra/?run=SRR11294037.

## Abbreviations

*IHH*: Indian Hedgehog
BDA-1: Brachydactyly type A1
BD: Brachydactyly
WES: Whole exome sequencing
WT: Wild type
MUT: Mutant
HSDS: Height Standard Deviation Score
HGMD: Human Gene Mutation Database
PTC: Patched Receptor
GATK: Genome Analysis Toolkit
SD: Standard Deviation
SMO: Smoothened
